# Adverse drug events and medication relation extraction in electronic health records with ensemble deep learning methods

**DOI:** 10.1093/jamia/ocz101

**Published:** 2019-08-07

**Authors:** Fenia Christopoulou, Thy Thy Tran, Sunil Kumar Sahu, Makoto Miwa, Sophia Ananiadou

**Affiliations:** 1 National Centre for Text Mining, School of Computer Science, The University of Manchester, Manchester, United Kingdom; 2 Artificial Intelligence Research Centre, National Institute of Advanced Industrial Science and Technology (AIST), Tokyo, Japan; 3 Toyota Technological Institute, Nagoya, Japan

**Keywords:** neural networks, adverse drug events, relation extraction, ensemble methods, electronic health records

## Abstract

**Objective:**

Identification of drugs, associated medication entities, and interactions among them are crucial to prevent unwanted effects of drug therapy, known as adverse drug events. This article describes our participation to the n2c2 shared-task in extracting relations between medication-related entities in electronic health records.

**Materials and Methods:**

We proposed an ensemble approach for relation extraction and classification between drugs and medication-related entities. We incorporated state-of-the-art named-entity recognition (NER) models based on bidirectional long short-term memory (BiLSTM) networks and conditional random fields (CRF) for end-to-end extraction. We additionally developed separate models for intra- and inter-sentence relation extraction and combined them using an ensemble method. The intra-sentence models rely on bidirectional long short-term memory networks and attention mechanisms and are able to capture dependencies between multiple related pairs in the same sentence. For the inter-sentence relations, we adopted a neural architecture that utilizes the Transformer network to improve performance in longer sequences.

**Results:**

Our team ranked third with a micro-averaged F1 score of 94.72% and 87.65% for relation and end-to-end relation extraction, respectively (Tracks 2 and 3). Our ensemble effectively takes advantages from our proposed models. Analysis of the reported results indicated that our proposed approach is more generalizable than the top-performing system, which employs additional training data- and corpus-driven processing techniques.

**Conclusions:**

We proposed a relation extraction system to identify relations between drugs and medication-related entities. The proposed approach is independent of external syntactic tools. Analysis showed that by using latent Drug-Drug interactions we were able to significantly improve the performance of non–Drug-Drug pairs in EHRs.

## INTRODUCTION

The interactions between drugs and medication-related entities are crucial to avoid harmful consequences of pharmaceuticals. In particular, adverse drug events (ADEs) reflect how much certain drugs can affect patients by causing undesirable side effects.^1^ Clinical narratives and electronic health records (EHRs) constitute a rich source for ADE evidence. Hence, careful examination of clinical narratives can provide helpful information for pharmacovigilance. However, the large amount of EHRs, as well as their informal and unstructured nature, makes the mining of interesting interactions related to ADEs a challenging task for clinicians. To tackle this issue, natural language processing (NLP) techniques have been widely applied on EHRs to automatically extract ADE-related information using relation extraction (RE) methods.

### Related work

Due to lack of publicly available data, initial approaches identified potential ADEs using co-occurrence statistics and feature-based methods, while evaluating on drugs with known adverse effects.^2^ Later, Kang et al^3^ built a knowledge base utilizing information from the Unified Medical Language System. Drugs and ADEs were determined based on a concept matching module. The shortest path between two concepts in the knowledge base was used to identify potential relations. Following feature-based techniques, graph topological and linguistic features were also explored to automatically detect drugs and their ADEs in unstructured text.^4^

Over the years, several researchers worked on creating additional annotated data with medication-drug interactions. The 2010 Informatics for Integrating Biology and the Bedside/Vetaran Affairs challenge on concepts, assertions, and relations in clinical text^5^ focused on RE among medical problem, treatment, and test pairs. The best performing systems in the challenge^6^^,^^7^ used dictionaries and feature-based methods, while a convolutional neural network (CNN) model was proposed to achieve competitive performance.^8^

A systematically annotated corpus was generated in Gurulingappa et al.^9^ for extraction of Drug-Dosage and Drug-*ADEs* relationships from medical case reports. Based on this corpus, an end-to-end system including CNN and bidirectional long-short term memory (BiLSTM) networks^10^ was proposed on the shortest dependency path of an entity pair.^11^ The method was extended by replacing the shortest dependency path with an attention mechanism,^12^ achieving higher performance. ADE relation extraction (RE) was treated as a multi-label problem using BiLSTMs in Bekoulis et al.^13^ Performance was further improved with adversarial training.^14^ Finally, Zhao et al^15^ treated ADEs relations as event structures, proposing a two-step event extraction process including CNNs and a beam search algorithm.

The 2017 Text Analysis Conference Adverse Reaction Extraction from Drug Labels Track 2^16^ asked participants to identify relations between adverse reactions and other named entities. The highest performing system in the challenge proposed a cascaded sequence labelling approach of BiLSTM conditional random fields (BiLSTM-CRF) networks for end-to-end RE^17^ while the second ranking system used BiLSTM-attention.^18^ A richer ADE-related corpus was developed by Munkhdalai et al,^19^ extending to 8 named entities and 7 relation types. They compared different models including support vector machine (SVM), LSTM and BiLSTM-attention. In the recent MADE (Medication, indication and Adverse Drug Events) 1.0 Challenge,^20^ participants had to identify relations between medication and ADEs, indications, other signs and symptoms. Once again, BiLSTM-attention networks achieved state-of-the-art performance.^21^^,^^22^

### Objective

In this work, we propose 3 neural network models to predict intra- and inter-sentence relations in EHRs as part of our participation in the 2018 n2c2 shared task on Challenges in Natural Language Processing for Clinical Data (https://n2c2.dbmi.hms.harvard.edu/track2). Our models are able to effectively extract relations between drugs and medication-related entities using BiLSTM-attention mechanisms and Transformer neural networks. Our contributions to the task mainly focus on RE models. In more detail, we introduce a walk-based model to support the identification of non–Drug-Drug pairs using inference chains between sentential entities.^23^ Our analysis showed that latent interactions between drugs in EHRs are particularly important to capture ADE-related associations. Additionally, since *ADE*-Drug pairs are often located several sentences apart,^20^ we propose a Transformer-based model to identify cross sentence relations. To the best of our knowledge, this is the first time Transformer is used for mention-level RE in clinical records. Our team ranked third in both relation and end-to-end extraction tasks. We report the submitted and improved performance of our models with in-depth analysis, showing the effectiveness of our methods to identify medication to drug relations in EHRs.

## MATERIALS AND METHODS

The n2c2 challenge (Track 2) aims to extract and classify drug-related interactions in EHRs. In particular, given an EHR with annotated drug and medication entities, the task requires the identification of potential interactions between them and their corresponding relation types. Based on the annotation scheme, the relation type between two entities can be formed as a combination of their semantic types. Hence, we treat this task as a binary classification problem and classify an entity pair as related or not. We propose intra- and inter-sentence neural models to identify relations of drugs with ADEs and other entities.

Motivated by the dynamics of different network layers, we first propose a weighted BiLSTM model that combines information from multiple neural layers, in contrast to existing models that use representations from the last neural layer only. Second, we aim to support the identification of related pairs using entity-based reasoning, in case context information is insufficient. We thus introduce a walk-based model that can infer entity pair associations using all existing entities in a sentence. In essence, the model can learn latent Drug-Drug interactions (DDIs) without any annotated data, to assist non-DDIs. To extract inter-sentence relations, we propose a Transformer-based network that can effectively memorize long-term dependencies.

### Intra-sentence models

To extract relations that reside in a single sentence, we developed two BiLSTM-based models following their reported effectiveness in similar tasks. Both models consider multiple entity pairs in a sentence, compared with existing state-of-the-art RE approaches that consider only one pair.^24^^,^^25^ The models have the same input and the first two architectural layers. The first model, named Weighted BiLSTM, aims to extract relation patterns that reside in the input sequence. The second model, named Walk-based model, is an extended version of the former, where a walk layer is stacked on top. It uses sentential entity graphs to infer relations between entities.

In the first layer (ie, the embedding layer), we map words, semantic entity types and relative positions to real-valued vectors. We follow the same approach as Zeng et al^26^ to represent the relative position of a word to the pair of interest, which we define as the target pair. We observe that in EHRs, several patterns express relations between entities without any supportive context words. For instance, the sentence *[itraconazole]_Drug_ [100mg]_Strength_ [qd]_Frequency_* is a typical example of a medical prescription, where no context words are present. Typically, the relations between *itraconazole* and attributes *100mg* and *qd* are inferred even without explicit textual evidence. As sequences of Drug*–*N number of non-Drug entities seem important, we combine word and entity-type information as the input representation of the network. This representation is then passed into a BiLSTM layer to encode sentential-context information into the word representations.

#### Weighted BiLSTM model

**Figure 1.**
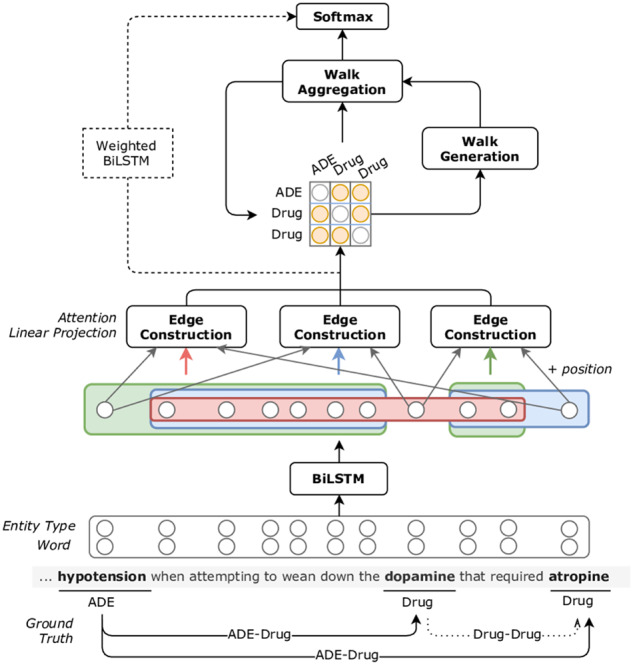
Architecture of the weighted bidirectional long-short term memory (Weighted BiLSTM) and Walk-based models.^24^ ADE: adverse drug event.

The Weighted BiLSTM model consists of four stacked layers as shown in Figure 1. This model aims to combine information from multiple neural layers to better represent a target pair. The word-based representations of each sentence are firstly fed into a two-stacked BiLSTM layer. We then combine the representations of the embedding layer and the output of the stacked BiLSTM into a weighted average, which results in context-aware word representations. We represent an entity by averaging its corresponding word representations. The new representations are augmented with relative position embeddings to the target entities and fed into an argument-based attention mechanism.^27^ The attention layer produces entity context representation based on the importance of the sentence words towards this entity. Finally, we form a final representation for each target pair by combining the representations of the target entities and their contexts. This pair representation is then fed into a binary classifier.

#### Walk-based model

The second intra-sentence model was first introduced for RE in the general domain. The model assumes that a potentially related entity pair can be supported by the relations between co-existing pairs in the same sentence. In the example sentence of Figure 1, the direct association between *hypotension* and *atropine* is not evident. However, if we use the *ADE-*Drug relation *hypotension-dopamine* and the Drug-Drug relation *dopamine-atropine,* the target association *hypotension-atropine* becomes clear. On that end, we restrict the generated pairs to include at least one drug, thus enabling DDIs. In fact, there are several DDIs in EHRs that can potentially affect the associations between drugs and ADEs.^28^ Although DDIs are not annotated in the n2c2 dataset, we use them as an intermediate step to infer non–Drug-Drug relations. Essentially, we infer the association between a pair using a series of interactions between entities in a sentence, including DDIs, as in the example shown in Figure 1.

To perform relation inference, we map a sentence into a directed graph structure, where entities constitute the nodes and edges correspond to the representation of the relation between two nodes. Figure 1 illustrates the proposed model, consisting of five layers. The initial edge representations of the entity graph (length L = 1) are equal to the entity pair representations, which are formed in the same way as in the Weighted BiLSTM model. We employ a two-step process, walk-generation and walk-aggregation (walk layer), to “walk” on the entity graph. By iterating N times, over the walk layer, we form walks of length up to 2^N^. Hence, we generate a finite number of walk representations using entity pairs from the first to the second target entity. These representations are merged into a final target pair representation using linear interpolation and fed into a binary classifier.

### Inter-sentence model

**Figure 2.**
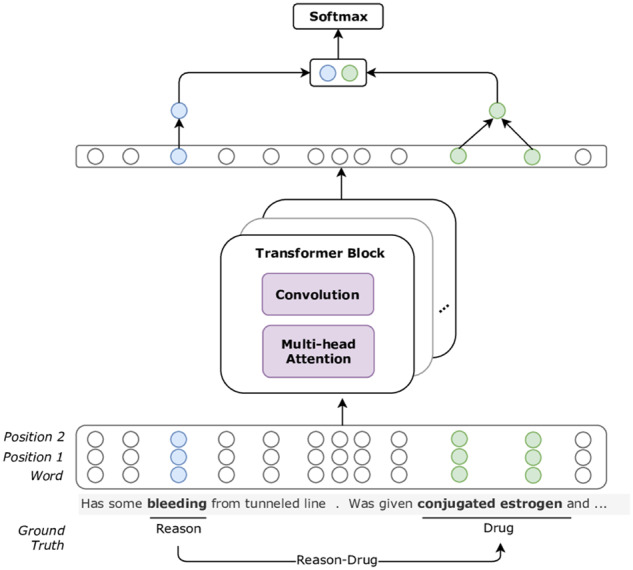
Model architecture of inter-sentence relation extraction utilizing the Transformer network.

In the n2c2 official training set, approximately 7% of relations are expressed across sentences. To explore cross-sentence interactions, we create relation candidates from multiple consecutive sentences. As represented in Figure 2, we employ the Transformer network.^27^ Transformer is a self-attention-based multilayer neural model that uses long word sequences to learn a new representation for each context word. One Transformer block constitutes of two subcomponents: (1) a multiheaded self-attention layer and (2) a position-wise feed-forward neural layer. Similar to Verga et al,^29^ we utilize a CNN with filter length equal to five in place of the feed-forward neural layer. To learn richer word representations, we stack multiple blocks with residual connections,^30^ named the Transformer layer.

Different from the intra-sentence models, the Transformer model treats a single target pair at a time. We generate relation candidates based on non-Drug and Drug pairs. Hence, we use the concatenation of word and relative position embeddings as input to the Transformer layer to form position-aware context representations. The output of the Transformer layer is a vector for each word of the input sequence. If a target entity has multiple tokens, we sum the token representations into a single embedding. To predict the relation of the target pair, we concatenate the embeddings of both target entities and feed them into a binary classifier.

### End-to-end Relation Extraction

To perform end-to-end RE, we build a pipeline system. We utilize the ensemble of state-of-the-art BiLSTM-CRF^31^ models and simpler feature-based CRF models for detection of named entities.^32^ The former model is able to recognize nested entities inside sentences which are essentially entities embedded into other entities. The latter model uses a set of different features, including orthographic, lexicosyntactic and clustering features.

### Experimental design

#### Data processing

The organizers provided 303 annotated discharge summaries extracted from MIMIC-III.^33^ We randomly split the documents into training and development sets (80% and 20%, respectively), while duplicate relations were ignored, as shown in Table 1. We used LingPipe for sentence splitting and OSCAR4 for word tokenization.^34^ We further split a sentence if it contains any of the following strings: “\n\n”, “:\n”, or “]\n”. If a token contains any of the following special characters “@, ? %) (”, we also broke it into fine-grained tokens. We additionally replaced terms that match the de-identified patient data such as “doctor X” or “patient X” with a static string of *DEIDTERM*, to reduce noise in the corpus.

**Table 1.** Statistics of n2c2 dataset in intra- and inter-sentence relations for the training and development sets

	Training		Development	
Total sentences	44 475		11 520	
Sentences with >1 entity	7125		1907	
Sentences with 1 entity	1835		401	
Sentences with no entities	35 515		9212	
Sentences with 1 pair	1672		409	
	**Inter**	**Intra**	**Inter**	**Intra**
Number of positive relations	1994	26591	570	7119
*Strength-Drug*	36	5276	13	1373
*Dosage-Drug*	107	3192	33	888
*Duration-Drug*	29	489	4	120
*Frequency-Drug*	158	4828	53	1259
*Form-Drug*	123	5060	74	1358
*Route-Drug*	107	4220	35	1173
*Reason-Drug*	1239	2830	307	783
*ADE-Drug*	195	696	51	165
Negative relations	78 471	39 850	20 050	9183
Duplicate relations	19		9	

ADE: adverse drug event.

#### Relation models and ensembling

We experimented with several settings for both intra- and inter-sentence models. For the Weighted BiLSTM model, we experimented with the number of stacked BiLSTM layers, attention and PubMed,^35^ or randomly initialized pretrained word embeddings. For the Walk-based model, we augmented the Weighted BiLSTM model with walks. We additionally experimented with different walk lengths, word embeddings and randomly removing non-related pairs in the training set, which we define as negative instance filtering. While training, negative filtering was used to counterbalance the bias towards the negative relation class. Finally, we concatenated consecutive sentences to perform inter-sentence RE. We generated instances containing up to three consecutive sentences as there is only 6% of relations across more than three sentences. We also examined different number of Transformer blocks.

To increase performance, we ensembled the relation models. In more detail, we generated intra-sentence relations using the Weighted BiLSTM and Walk-based models while predicting inter-sentence relations from the Inter-sentence model (when including intra-sentence relations from the inter-sentence model, performance was reduced). We tested simple ensemble techniques including majority^36^ and weighted voting.^37^ We finally selected majority voting as our ensembling method, as a result of higher performance on the development set. For each pair, we selected the prediction with dominating votes among models. Intra-sentence pairs predictions were collected from different intra-sentence models. For inter-sentence pairs, we selected predictions that resulted from the inter-sentence model alone. The combination of intra- and inter-sentence predicted pairs served as our final relation system predictions.

Our ensembling method included the fusion of several models. Specifically, we trained Walk-based models with different pretrained embeddings and walk lengths, as well as negative filtering inclusion/exclusion (on the best model), which resulted in 8 models. We additionally trained the highest recall setting 9 times using multiple initialization seeds. Concerning the Weighted BiLSTM model, we re-trained the best performing setting using 6 different hyper-parameter sets including gradient clipping, dropout rate, entity type and pair representation dimensions. Among intra-sentence ensembles, we selected the combination that provided the best performance on the development set. For the inter-sentence model, we trained 10 models with different initialization seeds. During training, we used early stopping on the development set to identify the best training epoch of each model. For evaluation on the test set, we retrained our models on the union of training and development sets.

#### End-to-end pipeline

We tuned the named-entity recognition (NER) components on the development set and selected 2 ensembles. One that provided the highest overall performance in terms of F1-score and another that had the largest recall, named “recall” NER. The second model enables more candidate pairs as it can predict more named entities. We trained the three proposed RE models on gold entities and during prediction, the output of the NER module was given as their input. Similarly to Track 2, for evaluation on the test set, we combined the training and development sets and used the best-performing ensemble on the development set.

## RESULTS

We report the performance of the proposed inter- and intra-sentential relation models on the development set in Table 2. We use the Approximate Randomization significance test^38^ to measure performance differences among models and settings. We consider statistical significance with *P* value <.05.

**Table 2.** Performance of the proposed RE models on the development set

Model	Precision	Recall	F1-score
	**Intra-sentence**		
Weighted, 1× BiLSTM	0.9702	0.8985	0.9330
+ Attention	0.9713	0.8975	0.9330
+ Walks L = 2	0.9767	0.9029	0.9384
+ Walks L = 4	0.9803	0.9040	0.9406
+ Walks L = 8	0.9804	0.9066	0.9420
+ Negative Filtering	0.9734	0.9115	0.9414
Weighted, 2× BiLSTM	0.9719	0.9057	0.9376
+ Attention	0.9722	0.9036	0.9366
	**Inter-sentence**		
sentence span 1, 2× Transformer	0.9549	0.9046	0.9291
sentence span 2, 1× Transformer	0.9265	0.8963	0.9112
sentence span 2, 2× Transformer	0.9358	0.9458	0.9408
sentence span 2, 3× Transformer	0.9240	0.9465	0.9351
sentence span 2, 4× Transformer	0.9198	0.9365	0.9281
sentence span 3, 2× Transformer	0.8851	0.9654	0.9235

BiLSTM: bidirectional long-short term memory; RE: relation extraction.

We tested the weighted model using different number of stacked layers and attention. The addition of attention did not significantly reduce the performance with one BiLSTM but it did for two-stacked. The main reason for this behavior may be that two-stacked layers capture fine-grained contextual information and therefore attention introduces noise. Significance testing between stacked BiLSTM layers showed that two-stacked layers significantly contribute to the performance.

We then stacked the walk layer on top of the single Weighted BiLSTM model. In the Walk-based model, we always consider attention to include explicit context information in the edges of the entity graph. We do not stack multiple BiLSTM layers while using walks to avoid over-parameterization. As observed, the Walk-based model achieves significantly better performance than the two-stacked BiLSTM with much less learned parameters. Furthermore, longer walks improve performance which resulted in our best performing model, including one weighted BiLSTM layer, attention and walks of L = 8. Significance testing between the walk models proved that longer walks are respectively better than shorter ones, in terms of F1-score. The performance slightly dropped when we applied negative filtering on top of the best walk model, but as expected, provided the highest recall among all intra-sentence models.

We finally tested the Transformer model on intra-sentence pairs (span 1). We observed that the model obtains significantly lower performance than the other two models. The best performance was achieved with 2-span sentences and 2 Transformer blocks, which is significantly better than using less or more blocks as well as longer sentence span. As most cross-sentence relations exist between two sentences (approximately 71%), introducing longer spans results in much more negative instances and consequently a highly imbalanced dataset.

Table 3 shows our submission and improved performance in the relation and end-to-end extraction tasks. Due to time limitations, the Walk-based model initially utilized a simple attention mechanism,^39^ as originally proposed in Christopoulou et al.^24^ However, additional experiments showed that argument-based attention yields better results (see Supplementary Appendix B). We further improved our intra-sentence ensemble by incorporating models with walk length less than L = 8. The new best ensemble model includes walks of length L = 2 and L = 8, 1 random seed model and 3 Weighted BiLSTM models. Statistical significance testing indicated that our new ensemble is significantly better than our submitted one in Track 2. For the end-to-end task, we used the best-performing pipeline on the development set. The submitted ensemble included the Weighted BiLSTM model alone and the “recall” NER ensemble. When we used our improved RE model on the output of our “recall” NER performance improved.

**Table 3.** Performance on test set for relation (Track 2) and end-to-end (Track 3) extraction task of submitted and improved models.

Model	Precision	Recall	F1-score
	**Relation**		
* Intra [ensemble] + Inter [ensemble]	0.9463	0.9480	0.9472
Intra [ensemble] + Inter [ensemble]	0.9572	0.9456	0.9514
	**End-to-end**		
* NER [recall] + Weighted [ensemble] + Inter [ensemble]	0.9264	0.8318	0.8765
NER [recall] + Intra [ensemble] + Inter [ensemble]	0.9286	0.8321	0.8777

The asterisk indicates our submitted models to the n2c2 shared task.

NER: named entity recognition.

## DISCUSSION

### Error analysis

Because we treat this task as a binary classification problem, errors are restricted to two classes. Additionally, there are no directionality errors as the relation is always from a non-Drug to a Drug entity. We analyze the incorrect predictions of our models using category-wise false positive rates (FPR) and false negative rate (FNR). We estimate the error rate as the proportion of all negative instances that were misclassified as positive (FPR) and the proportion of all positive instances that were misclassified as negative (FNR), as shown in Equations 1 and 2,

(1) $$\mathrm{FP}R_{i}=\frac{\#\;\mathrm{FP}\;\mathrm{in}\;\mathrm{class}\;i}{\#\;\mathrm{FP}\;\mathrm{in}\;\mathrm{class}\;i\;+\;\#\;\mathrm{TN}\;\mathrm{in}\;\mathrm{class}\;i}$$

(2) $$\mathrm{FN}R_{i}=\frac{\#\mathrm{FN}\;\mathrm{in}\;\mathrm{class}\;i}{\#\mathrm{FN}\;\mathrm{in}\;\mathrm{class}\;i\;+\#\;\mathrm{TP}\;\mathrm{in}\;\mathrm{class}\;i}$$

**Figure 3.**
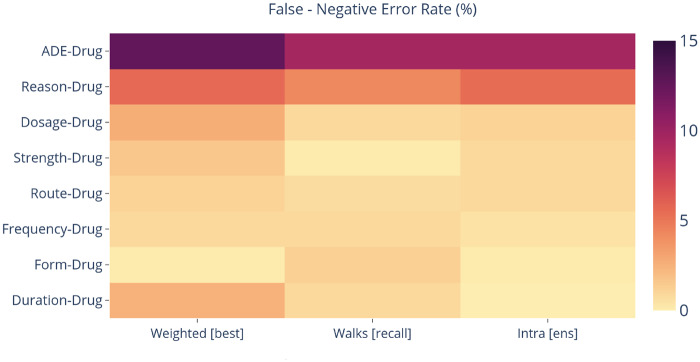
False negative error rate of intra-sentence models and their ensemble on the development set. ADE: adverse drug event.

Figure 3 visualizes the false negative error rates of our intra-sentence models and their ensemble, as evaluated only on intra-sentence pairs (we do not report the FPR, as we found it was below 1% for all models and relation classes) (Supplementary Appendix C). It is observed that *ADE-*Drug and *Reason*-Drug classes have the highest probability to misclassify a pair as negative (10% for *ADE* and 5% for *Reason*). In fact, these classes are the most difficult to predict, as they require well-formed context and relation-indicative words. In the sentence “Allergies: *[Bactrim]_Drug_* (*[rash]_ADE_*),” the relation between *ADE* and Drug is not evident as there are no keywords to support it. In contrast, Duration, Form, Strength, and other similar entities are always found close to a drug and follow a standard pattern which can be learned from sequential models eg, “*[Azithromycin]_Drug_ [250 mg]_Strength_ [Tablet Sig]_Form_”.* Although Duration-Drug has the least positive occurrences in the dataset, our models can detect it since it is always related to the closest drug. Compared with Weighted BiLSTM, the Walk-based model is less biased to negative relations, as the introduction of negative filtering and the walk-inference enables the identification of more positive instances. The combination of models reduces the FNR. As we did not develop category-wise classifiers, the models try to fit all relation patterns under a single category. Because *ADE-* and Reason-Drug patterns are much less, compared with other non–Drug-Drug pairs, all models tend to have lower performance on these particular categories.

### Relative contributions

**Figure 4.**
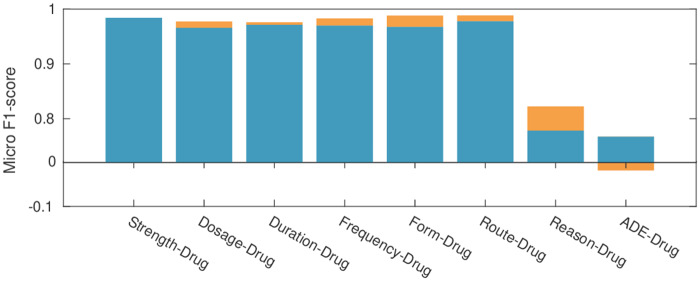
Performance of the best relation extraction ensemble on each relation class on the development set. Blue bars indicate the performance of the intra-sentence model ensemble (Walk and Weighted models), while orange bars show performance improvement when merging intra- and inter-sentence models. ADE: adverse drug event.

Next, we investigate the contribution of the inter-sentence model to the overall performance. Figure 4 illustrates our best intra-sentence ensemble and the improvement after merging with inter-sentence predictions. As expected, the Reason-Drug class has the highest improvement due to the large amount of inter-sentence relations in the dataset (62% of intersentence relations). However, *ADE-*Drug performance drops despite the number of cross-sentence *ADE-*Drug pairs (10%), as Transformer fails to detect them. This is due to the semantic structure of these pairs, which, in most cases, require logical inference from one sentence to the other (eg, “*The patient had [two transfusion reactions]_ADE_ to [platelets]_Drug_ when first admitted. She was premedicated with [anti-histamines]_Drug_*”). The relation between *two transfusion reactions* and *anti-histamines* is inferred based on implied context, not present in the snippet.

**Figure 5.**
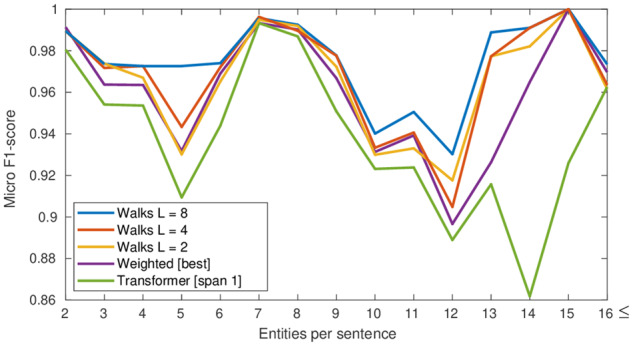
Performance of intra-sentence models on the development set on sentences with different number of entities.

We then analyze the importance of the walk layer by measuring the performance on sentences with several entities. As shown in Figure 5, performance increases with longer walks. Among walk lengths, L = 8 has the best performance across multi-entity sentences, outperforming the other two models. This indicates that graph-based methods can be helpful for RE.

We further investigate the contribution of DDIs in the walks model. We retrain Walk-based models without DDIs and by considering only non–Drug-Drug pairs when forming walks. In this setting, the *ADE-*Drug pair *hypotension*-*atropine* of Figure 1, cannot incorporate walks of L = 2 in its representation, as valid entity paths between the corresponding target entities cannot be formed. In essence, by removing DDIs, we restrict the relation inference steps between two entities. As it is noticed from Table 4, the Walk-based model performs significantly lower without DDIs. Additionally, significance testing designated that different walk lengths perform similarly when excluding DDIs. All these observations indicate the importance of latent DDIs in inferring other related pairs.

**Table 4.** Performance of the Walk-based model with and without considering Drug-Drug pairs

F1-score	Exclude DDIs			Include DDIs		
	L = 8	L = 4	L = 2	L = 8	L = 4	L = 2
Micro	0.9366	0.9366	0.9366	0.9420	0.9406	0.9384
Macro	0.9345	0.9345	0.9335	0.9389	0.9367	0.9349

DDI: Drug-Drug interaction.

### Performance comparison

We finally compare our models with the best performing systems in relation and end-to-end tracks. Regarding Track 2, the top-ranking team utilized a joint approach, achieving a micro-averaged F1-score of 96.3%. However the predicted relations were post-processed with heuristics: addition of relations between isolated attributes and their nearest drugs. Considering non-post-processed predictions, their system achieved 93.99% in micro F1-score^40^, which is evidently lower than our submitted performance of 94.72% and our improved model of 95.14% (we implemented the same postprocessing rule, but we could not get higher performance). The same team ranked first in the end-to-end extraction track. The second best performing system in Track 3 used additional training data for NER, as well as information from the MIMIC-III and SIDER databases.^41^ According to the organizers, there is no significant difference between their system performance and ours.

## CONCLUSION

In this work, we proposed an ensemble method for RE between drugs and medications, as part of our participation to the n2c2 challenge 2018. Our models ranked on the third place in both relation (Track 2) and end-to-end extraction (Track 3).

We described three deep neural models that are independent of external syntactic and linguistic tools. A Weighted BiLSTM and a Walk-based model were introduced for extracting relations in EHRs. The proposed Walk-based model is able to infer associations between entities by taking advantage of co-existing entity pairs in the same sentence. Further exploration indicated that latent DDIs are particularly important to infer non–Drug-Drug associations. We also presented a Transformer-based network for mention-level RE. The approach we follow in this work utilizes much fewer parameters than the originally proposed network. Analysis of the top-performing systems showed that our approach can achieve comparable performance without additional training data and post-processing rules.

The proposed models can be applied to any RE task, to identify relations between biomedical or generic domain named entities. Due to the low performance of our models on *ADE*- and Reason-Drug categories, we aim, as future work, to further investigate these interactions by speculating their linguistic properties and develop more suitable models. We also intend to exploit joint-learning approaches for end-to-end RE.^42^ Finally, we plan to apply the proposed approach to other biomedical RE corpora, as well as to collaborate with clinicians, to show not only the clinical significance of our methods but also their generalizability.

## FUNDING

This research was supported with funding from Biotechnology and Biological Services Research Council EMPATHY (Enriching Metabolic PATHwaY) models with evidence from the literature (Grant ID: BB/M006891/1) (SA) and the Manchester Molecular Pathology Innovation Centre (Grant ID: MR/N00583X/1) (SA).

## AUTHOR CONTRIBUTIONS

The Walk-based, Weighted BiLSTM, and Transformer models were implemented by FC, TTT, and SKS, respectively. TTT implemented the model ensembling. FC and TTT conducted error analysis and drafted the manuscript. MM and SA supervised all steps of the work. SKS, MM, and SA revised the manuscript. All authors provided feedback and read and approved the final version of the manuscript.

## Supplementary Material

ocz101_Supplementary_Data
