# Supplementary material for: Adverse drug events and medication relation extraction in electronic health records with ensemble deep learning methods
Source: J Am Med Inform Assoc. 2019 Aug 7;27(1):39–46. doi: 10.1093/jamia/ocz101 (PMC6913215; doi:10.1093/jamia/ocz101)
Supplement: ocz101_Supplementary_Data [file ocz101_supplementary_data.docx]

## APPENDIX A. HYPER-PARAMETER SETTINGS

The Weighted BiLSTM model was implemented using Theano[1], the Walk-based model was implemented using Chainer[2] and the Transformer model was based on Tensorflow[3]. Table 5 shows the parameter range that we used to tune our models on the development set.

Table 5. Deep neural models hyper-parameters and the range used for tuning the intra-sentence models.

| Parameter | Range |
| --- | --- |
| Position dimension | [10, 50] |
| Entity type dimension | [10, 32] |
| Final embedding dimension | [50, 100] |
| Attention mechanism | simple attention, scale dot attention |
| Dropout | [0.01, 0.3] |
| L2 regularisation | [10^-6^,10 ^-1^] |
| Gradient clipping | [5, 30] |
| Walk length | [1, 2, 4, 8] |
| Pre-trained word embedding | PubMed, random initialisation |

## APPENDIX B. ADDITIONAL RESULTS

We report additional results on Track 2, relation extraction, on the development and test sets in Tables 6-10. Table 11 reports the category-wise performance on Track 3, end-to-end relation extraction on the test set.

Table 6. Best submitted performance on development and test set for Track 2, relation extraction.

| Category | Development | | | Test | | |
| --- | --- | --- | --- | --- | --- | --- |
|  | **Precision** | **Recall** | **F1-score** | **Precision** | **Recall** | **F1-score** |
| Strength-Drug | 0.9899 | 0.9913 | 0.9906 | 0.9879 | 0.9840 | 0.9860 |
| Dosage-Drug | 0.9838 | 0.9848 | 0.9843 | 0.9814 | 0.9777 | 0.9796 |
| Duration-Drug | 0.9524 | 0.9677 | 0.9600 | 0.8968 | 0.9178 | 0.9072 |
| Frequency-Drug | 0.9885 | 0.9855 | 0.9870 | 0.9772 | 0.9655 | 0.9713 |
| Form-Drug | 0.9882 | 0.9882 | 0.9882 | 0.9921 | 0.9792 | 0.9856 |
| Route-Drug | 0.9933 | 0.9835 | 0.9884 | 0.9804 | 0.9729 | 0.9766 |
| Reason-Drug | 0.8737 | 0.7885 | 0.8289 | 0.8080 | 0.8182 | 0.8131 |
| ADE-Drug | 0.7252 | 0.7454 | 0.7352 | 0.7029 | 0.8458 | 0.7678 |
| Micro | 0.9656 | 0.9516 | 0.9586 | 0.9462 | 0.9479 | **0.9472** |
| Macro | 0.9599 | 0.9463 | 0.9517 | 0.9408 | 0.9449 | **0.9420** |

Table 7. Improved performance on development and test set for Track 2, relation extraction.

| Category | Development | | | Test | | |
| --- | --- | --- | --- | --- | --- | --- |
|  | **Precision** | **Recall** | **F1-score** | **Precision** | **Recall** | **F1-score** |
| Strength-Drug | 0.9856 | 0.9863 | 0.9859 | 0.9875 | 0.9828 | 0.9851 |
| Dosage-Drug | 0.9805 | 0.9816 | 0.9811 | 0.9835 | 0.9766 | 0.9801 |
| Duration-Drug | 0.9606 | 0.9839 | 0.9721 | 0.9279 | 0.9061 | 0.9169 |
| Frequency-Drug | 0.9863 | 0.9871 | 0.9867 | 0.989 | 0.9633 | 0.9760 |
| Form-Drug | 0.9882 | 0.9875 | 0.9878 | 0.9935 | 0.9866 | 0.9901 |
| Route-Drug | 0.9925 | 0.9859 | 0.9892 | 0.9854 | 0.9701 | 0.9777 |
| Reason-Drug | 0.8673 | 0.7803 | 0.8215 | 0.8386 | 0.8061 | 0.8220 |
| ADE-Drug | 0.7111 | 0.7407 | 0.7256 | 0.7406 | 0.8295 | **0.7825** |
| Micro | 0.9628 | 0.9498 | **0.9562** | 0.9572 | 0.9456 | **0.9514** |
| Macro | 0.9556 | 0.9452 | 0.9491 | 0.9549 | 0.9463 | 0.9497 |

Table 8. Category-wise performance in terms of F1-score for intra-sentence relation extraction models on the development set. Inter (span=1) refers to the inter-sentence Transformer model, when used it in intra-sentence settings.

| **Category** | **Walks (recall)** | **Walks (best)** | **Weighted (best)** | **Inter (span=1)** |
| --- | --- | --- | --- | --- |
| Strength-Drug | 0.9848 | 0.9877 | 0.9786 | 0.9704 |
| Dosage-Drug | 0.9653 | 0.9652 | 0.9542 | 0.9623 |
| Duration-Drug | 0.9520 | 0.9633 | 0.9590 | 0.9398 |
| Frequency-Drug | 0.9709 | 0.9724 | 0.9659 | 0.9602 |
| Form-Drug | 0.9655 | 0.9658 | 0.9658 | 0.9621 |
| Route-Drug | 0.9769 | 0.9760 | 0.9748 | 0.9651 |
| Reason-Drug | 0.7774 | 0.7730 | 0.7742 | 0.7488 |
| ADE-Drug | 0.7469 | 0.7379 | 0.7385 | 0.7112 |
| **Micro** | 0.9414 | 0.9420 | 0.9376 | 0.9291 |
| **Macro** | 0.9378 | 0.9389 | 0.9342 | 0.9276 |

Table 9. Performance of the Walk-based model on the development set in terms of micro-averaged F1-score, for different walk lengths, attention mechanisms and pre-trained word embeddings. PubMed and Random indicate the usage of pre-trained word embeddings and randomly initialized word embeddings, respectively. NF indicates the addition of Negative Filtering.

| Model | PubMed | | | Random | | |
| --- | --- | --- | --- | --- | --- | --- |
|  | **Precision** | **Recall** | **F1-score** | **Precision** | **Recall** | **F1-score** |
|  | **Simple Attention** | | | | | |
| L2 | 0.9715 | 0.8990 | 0.9339 | 0.9656 | 0.8879 | 0.9251 |
| L4 | 0.9746 | 0.9027 | 0.9373 | 0.9776 | 0.8990 | 0.9367 |
| L8 | 0.9751 | 0.9029 | 0.9376 | 0.9781 | 0.9051 | 0.9402 |
| L8 + NF | 0.9711 | 0.9105 | 0.9398 | 0.9737 | 0.9088 | 0.9401 |
|  | **Argument-based Attention** | | | | | |
| L2 | 0.9753 | 0.9033 | 0.9380 | 0.9767 | 0.9029 | 0.9384 |
| L4 | 0.9800 | 0.9051 | 0.9411 | 0.9803 | 0.9040 | 0.9406 |
| L8 | 0.9787 | 0.8994 | 0.9374 | 0.9804 | 0.9066 | 0.9420 |
| L8 + NF | 0.9713 | 0.9105 | 0.9399 | 0.9734 | 0.9115 | 0.9414 |

Table 10. Performance of the Walk-based model with and without *Drug*-*Drug* pairs on different relation classes. As it is observed, long walks (L=8) significantly improve the performance of *Reason*-*Drug* pairs by almost 2 percentage points.

| **Category** | ***Exclude* DDIs** | | | ***Include* DDIs** | | |
| --- | --- | --- | --- | --- | --- | --- |
|  | **L=8** | **L=4** | **L=2** | **L=8** | **L=4** | **L=2** |
| Strength-Drug | 0.9869 | 0.9866 | 0.9869 | 0.9877 | 0.9866 | 0.9869 |
| Dosage-Drug | 0.9609 | 0.9587 | 0.9593 | 0.9652 | 0.9636 | 0.9641 |
| Duration-Drug | 0.9630 | 0.9672 | 0.9633 | 0.9633 | 0.9547 | 0.9551 |
| Frequency-Drug | 0.9621 | 0.9602 | 0.9601 | 0.9724 | 0.9692 | 0.9670 |
| Form-Drug | 0.9638 | 0.9626 | 0.9633 | 0.9658 | 0.9654 | 0.9640 |
| Route-Drug | 0.9711 | 0.9703 | 0.9715 | 0.9760 | 0.9751 | 0.9744 |
| Reason-Drug | 0.756 | 0.7620 | 0.7556 | 0.7730 | 0.7689 | 0.7574 |
| ADE-Drug | 0.7443 | 0.7500 | 0.7462 | 0.7379 | 0.7423 | 0.7323 |
| **Micro** | 0.9366 | 0.9366 | 0.9360 | 0.9420 | 0.9406 | 0.9384 |
| **Macro** | 0.9345 | 0.9345 | 0.9335 | 0.9389 | 0.9367 | 0.9349 |

Table 11. Best submitted and improved performance on test set for Track 3, end-to-end relation extraction.

| Category | Submitted | | | Improved | | |
| --- | --- | --- | --- | --- | --- | --- |
|  | **Precision** | **Recall** | **F1-score** | **Precision** | **Recall** | **F1-score** |
| Strength-Drug | 0.9740 | 0.9621 | 0.9680 | 0.9780 | 0.9651 | 0.9715 |
| Dosage-Drug | 0.9311 | 0.9124 | 0.9217 | 0.9336 | 0.9139 | 0.9236 |
| Duration-Drug | 0.7983 | 0.6784 | 0.7335 | 0.8319 | 0.6737 | 0.7445 |
| Frequency-Drug | 0.9672 | 0.9286 | 0.9475 | 0.9681 | 0.9253 | 0.9462 |
| Form-Drug | 0.9588 | 0.9264 | 0.9423 | 0.9597 | 0.9316 | 0.9454 |
| Route-Drug | 0.9516 | 0.914 | 0.9324 | 0.9573 | 0.9114 | 0.9338 |
| Reason-Drug | 0.7179 | 0.4463 | 0.5505 | 0.7122 | 0.4435 | 0.5466 |
| ADE-Drug | 0.5020 | 0.1678 | 0.2515 | 0.5039 | 0.1746 | 0.2594 |
| Micro | 0.9264 | 0.8318 | **0.8765** | 0.9286 | 0.8321 | 0.8777 |
| Macro | 0.9072 | 0.7954 | 0.8423 | 0.9081 | 0.7966 | 0.8435 |

In table 12, we report the performance of the top participating teams in Tracks 2 and 3. Our improved model achieves comparable performance with the 2nd team in Track 2, as well as outperforms the 1st team without utilizing post-processing techniques. The same applies to the end-to-end relation extraction task as our improved score is very close to the performance of the 1st team, without post-processing.

Table 12. Reported performance of the top-3 performing systems in relation extraction and end-to-end tasks. Asterisk *, indicates that there is no significant difference between the 2nd team model and our submitted end-to-end models - reported by the shared task organizers. While only considering original predictions without post-processing, our model performed competitive to the 1st team in the relation extraction task.

| Task | Rank | Method | F1-score | |
| --- | --- | --- | --- | --- |
|  |  |  | **Origin** | **Post-processing** |
| Relation Extraction | 1 | UTHealth/Dalian | 0.9399 | 0.9630 |
|  | 2 | VA Salt Lake City / University of Utah | 0.9530 | - |
|  | 3 | Ours (submitted) | 0.9472 | - |
|  |  | Ours (improved) | 0.9514 | - |
| End-to-End | 1 | UTHealth/Dalian | 0.8792 | 0.8905 |
|  | 2 | University of Florida | 0.8778* | - |
|  | 3 | Ours (submitted) | 0.8765* | - |
|  |  | Ours (improved) | 0.8777 | - |

##

## APPENDIX C. STATISTICS

Table 13 illustrates category-wise False Positive and False Negative rates for intra-sentence models and their ensemble.

Table 13. False positive (FPR) and False negative (FNR) rate for intra-sentence models on the development set.

| Category | FPR (%) | | | FNR (%) | | | |
| --- | --- | --- | --- | --- | --- | --- | --- |
|  | **Weighted**  **(best)** | **Walks**  **(recall)** | **Intra (ensemble)** | **Weighted (best)** | | **Walks**  **(recall)** | **Intra (ensemble)** |
| Strength-Drug | 0.02 | 0.02 | 0.02 | 1.60 | | 0.87 | 0.87 |
| Dosage-Drug | 0.04 | 0.03 | 0.02 | 2.70 | | 1.24 | 1.13 |
| Duration-Drug | 0.03 | 0.08 | 0.04 | 2.50 | | 0.83 | 0.00 |
| Frequency-Drug | 0.02 | 0.01 | 0.01 | 0.87 | | 0.71 | 0.48 |
| Form-Drug | 0.01 | 0.01 | 0.01 | 0.15 | | 0.15 | 0.15 |
| Route-Drug | 0.01 | 0.01 | 0.01 | 1.11 | | 0.85 | 0.85 |
| Reason-Drug | 0.13 | 0.14 | 0.12 | 5.62 | | 4.34 | 5.49 |
| ADE-Drug | 0.21 | 0.23 | 0.22 | 12.73 | | 9.70 | 9.70 |

|  |  |  |
| --- | --- | --- |

| **REFERENCES** |  |  |
| --- | --- | --- |

1. Al-Rfou R, Alain G, Almahairi A, et al. Theano: a Python framework for fast computation of mathematical expressions. arXiv 2016 May 9 [E-pub ahead of print].
2. Tokui S, Oono K, Hido S, et al. Chainer: a next-generation open source framework for deep learning. In: Proceedings of LearningSys in NIPS; 2015; (5): 1–6.
3. Abadi M, Agarwal A, Barham P, et al. TensorFlow: large-scale machine learning on heterogeneous systems. In: Proceedings of OSDI; 2016; 265–83.
